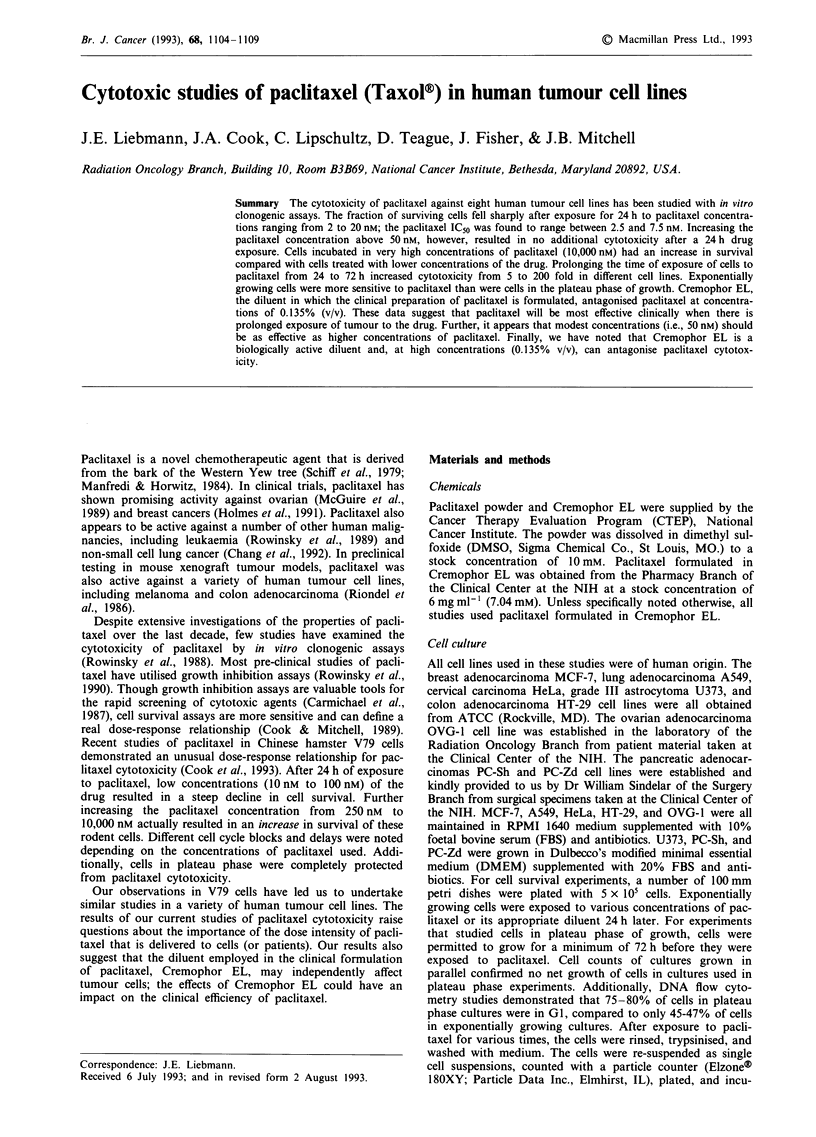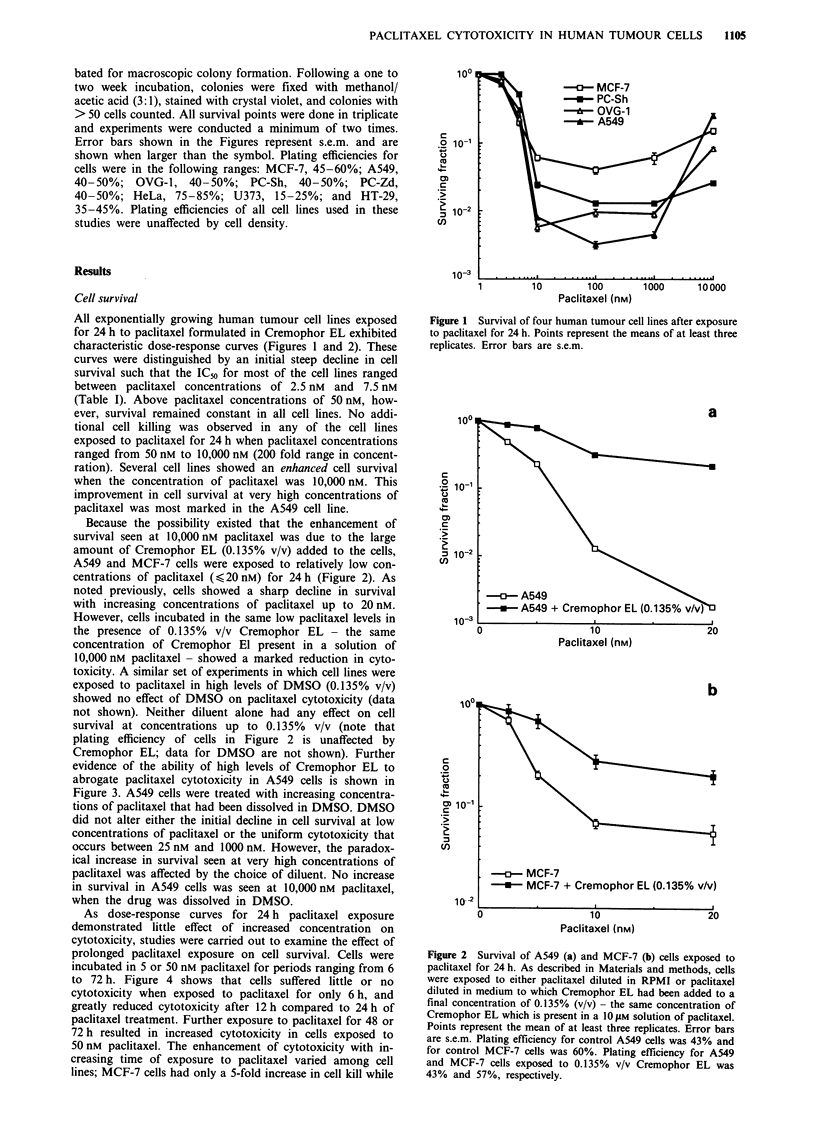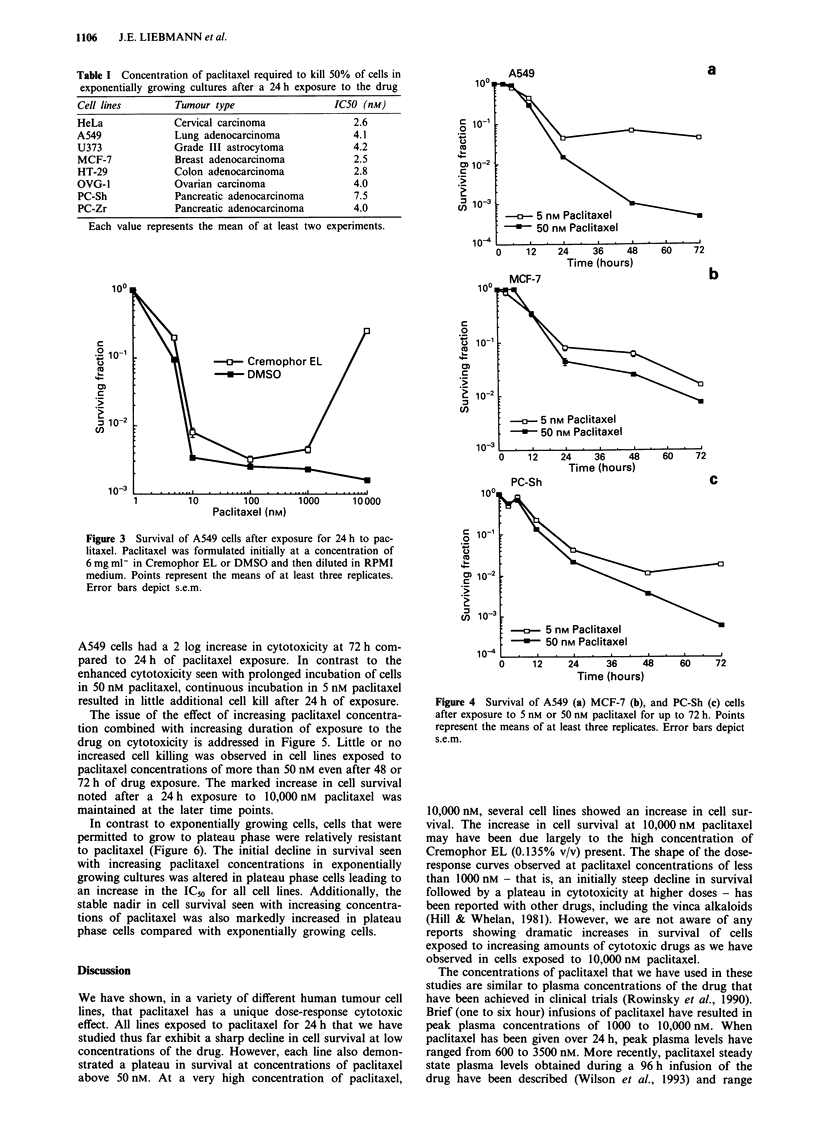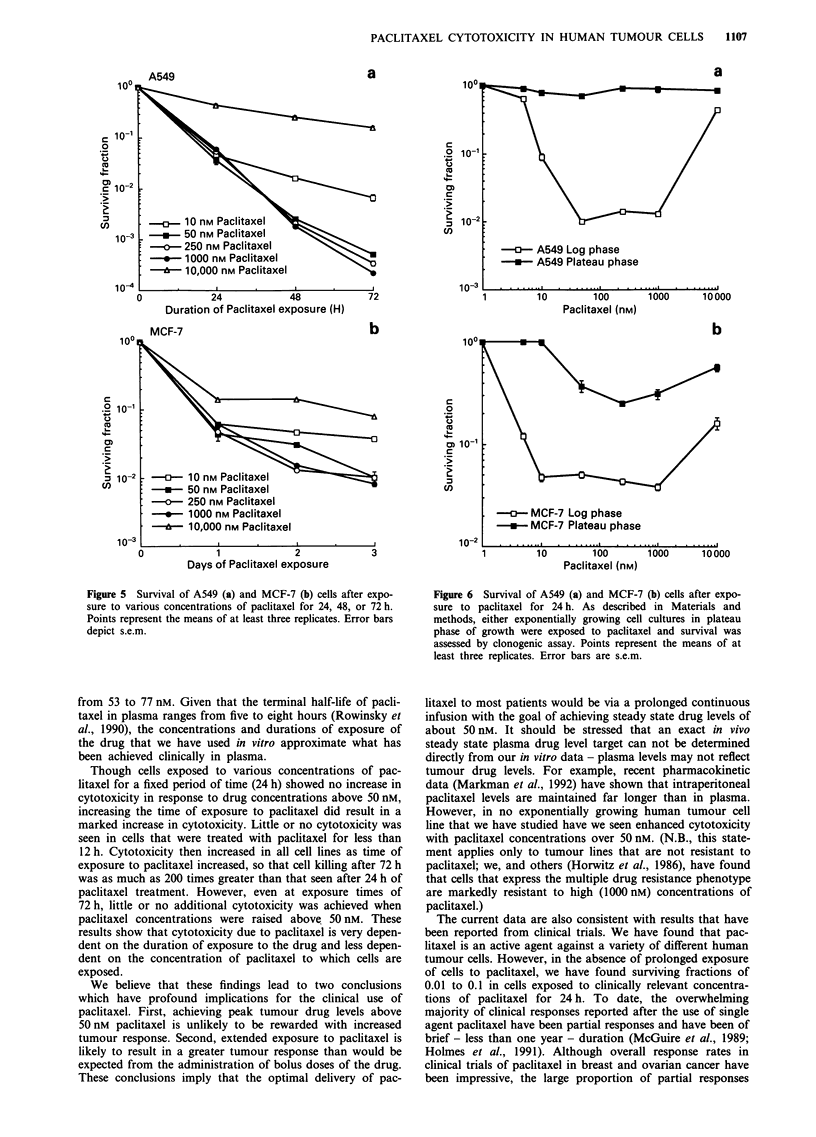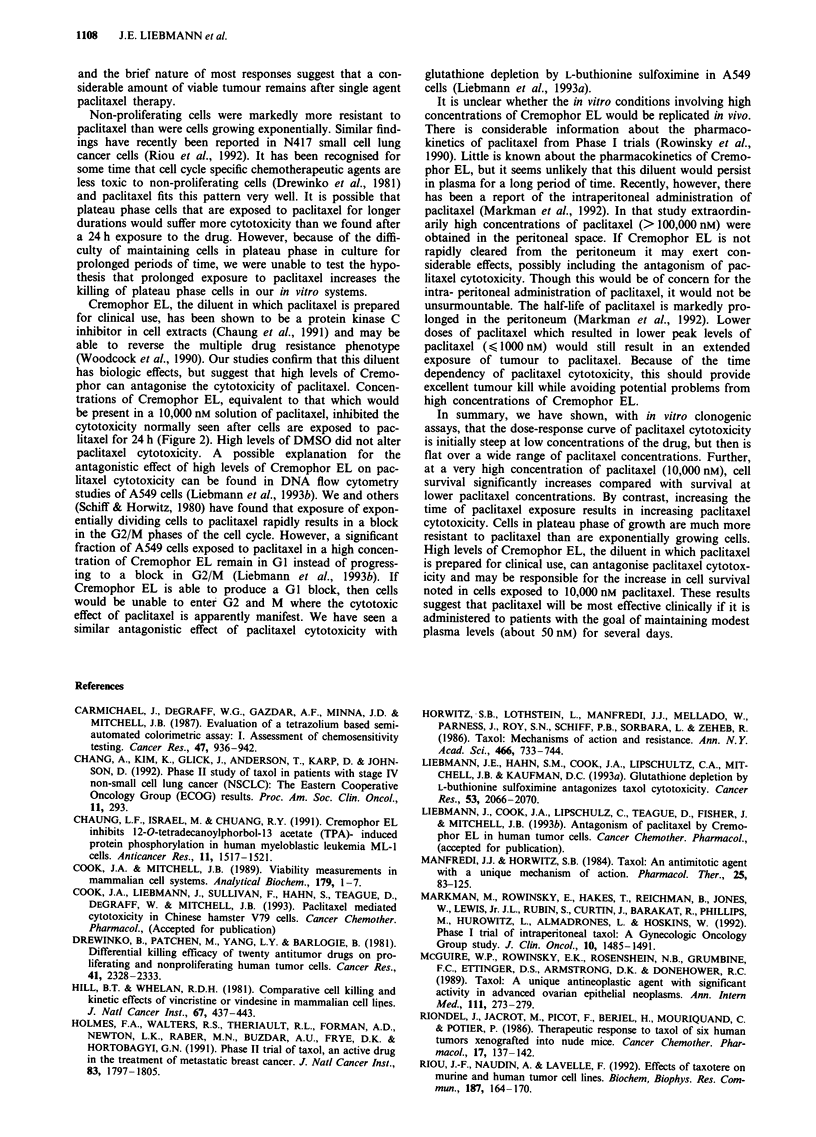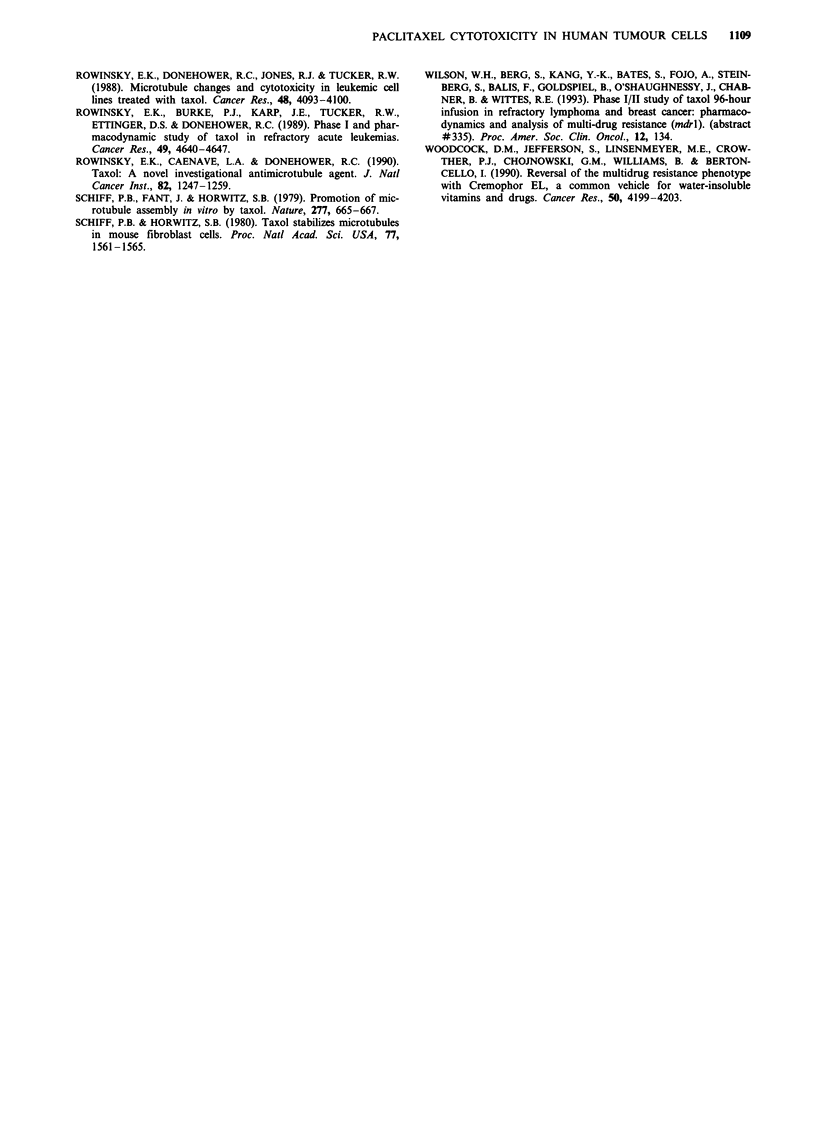# Cytotoxic studies of paclitaxel (Taxol) in human tumour cell lines.

**DOI:** 10.1038/bjc.1993.488

**Published:** 1993-12

**Authors:** J. E. Liebmann, J. A. Cook, C. Lipschultz, D. Teague, J. Fisher, J. B. Mitchell

**Affiliations:** Radiation Oncology Branch, National Cancer Institute, Bethesda, Maryland 20892.

## Abstract

The cytotoxicity of paclitaxel against eight human tumour cell lines has been studied with in vitro clonogenic assays. The fraction of surviving cells fell sharply after exposure for 24 h to paclitaxel concentrations ranging from 2 to 20 nM; the paclitaxel IC50 was found to range between 2.5 and 7.5 nM. Increasing the paclitaxel concentration above 50 nM, however, resulted in no additional cytotoxicity after a 24 h drug exposure. Cells incubated in very high concentrations of paclitaxel (10,000 nM) had an increase in survival compared with cells treated with lower concentrations of the drug. Prolonging the time of exposure of cells to paclitaxel from 24 to 72 h increased cytotoxicity from 5 to 200 fold in different cell lines. Exponentially growing cells were more sensitive to paclitaxel than were cells in the plateau phase of growth. Cremophor EL, the diluent in which the clinical preparation of paclitaxel is formulated, antagonised paclitaxel at concentrations of 0.135% (v/v). These data suggest that paclitaxel will be most effective clinically when there is prolonged exposure of tumour to the drug. Further, it appears that modest concentrations (i.e., 50 nM) should be as effective as higher concentrations of paclitaxel. Finally, we have noted that Cremophor EL is a biologically active diluent and, at high concentrations (0.135% v/v), can antagonise paclitaxel cytotoxicity.


Br. J. Cancer (1993), 68, 1104–1109 © Macmillan Press Ltd., 1993

### Cytotoxic studies of paclitaxel (Taxol®) in human tumour cell lines

J.E. Liebmann, J.A. Cook, C. Lipschultz, D. Teague, J. Fisher, & J.B. Mitchell

*Radiation Oncology Branch, Building 10, Room B3B69, National Cancer Institute, Bethesda, Maryland 20892, USA.*

**Summary** The cytotoxicity of paclitaxel against eight human tumour cell lines has been studied with *in vitro* clonogenic assays. The fraction of surviving cells fell sharply after exposure for 24 h to paclitaxel concentrations ranging from 2 to 20 nM; the paclitaxel $IC_{50}$ was found to range between 2.5 and 7.5 nM. Increasing the paclitaxel concentration above 50 nM, however, resulted in no additional cytotoxicity after a 24 h drug exposure. Cells incubated in very high concentrations of paclitaxel (10,000 nM) had an increase in survival compared with cells treated with lower concentrations of the drug. Prolonging the time of exposure of cells to paclitaxel from 24 to 72 h increased cytotoxicity from 5 to 200 fold in different cell lines. Exponentially growing cells were more sensitive to paclitaxel than were cells in the plateau phase of growth. Cremophor EL, the diluent in which the clinical preparation of paclitaxel is formulated, antagonised paclitaxel at concentrations of 0.135% (v/v). These data suggest that paclitaxel will be most effective clinically when there is prolonged exposure of tumour to the drug. Further, it appears that modest concentrations (i.e., 50 nM) should be as effective as higher concentrations of paclitaxel. Finally, we have noted that Cremophor EL is a biologically active diluent and, at high concentrations (0.135% v/v), can antagonise paclitaxel cytotoxicity.

Paclitaxel is a novel chemotherapeutic agent that is derived from the bark of the Western Yew tree (Schiff *et al.*, 1979; Manfredi & Horwitz, 1984). In clinical trials, paclitaxel has shown promising activity against ovarian (McGuire *et al.*, 1989) and breast cancers (Holmes *et al.*, 1991). Paclitaxel also appears to be active against a number of other human malignancies, including leukaemia (Rowinsky *et al.*, 1989) and non-small cell lung cancer (Chang *et al.*, 1992). In preclinical testing in mouse xenograft tumour models, paclitaxel was also active against a variety of human tumour cell lines, including melanoma and colon adenocarcinoma (Riondel *et al.*, 1986).

Despite extensive investigations of the properties of paclitaxel over the last decade, few studies have examined the cytotoxicity of paclitaxel by *in vitro* clonogenic assays (Rowinsky *et al.*, 1988). Most pre-clinical studies of paclitaxel have utilised growth inhibition assays (Rowinsky *et al.*, 1990). Though growth inhibition assays are valuable tools for the rapid screening of cytotoxic agents (Carmichael *et al.*, 1987), cell survival assays are more sensitive and can define a real dose-response relationship (Cook & Mitchell, 1989). Recent studies of paclitaxel in Chinese hamster V79 cells demonstrated an unusual dose-response relationship for paclitaxel cytotoxicity (Cook *et al.*, 1993). After 24 h of exposure to paclitaxel, low concentrations (10 nM to 100 nM) of the drug resulted in a steep decline in cell survival. Further increasing the paclitaxel concentration from 250 nM to 10,000 nM actually resulted in an *increase* in survival of these rodent cells. Different cell cycle blocks and delays were noted depending on the concentrations of paclitaxel used. Additionally, cells in plateau phase were completely protected from paclitaxel cytotoxicity.

Our observations in V79 cells have led us to undertake similar studies in a variety of human tumour cell lines. The results of our current studies of paclitaxel cytotoxicity raise questions about the importance of the dose intensity of paclitaxel that is delivered to cells (or patients). Our results also suggest that the diluent employed in the clinical formulation of paclitaxel, Cremophor EL, may independently affect tumour cells; the effects of Cremophor EL could have an impact on the clinical efficiency of paclitaxel.

Correspondence: J.E. Liebmann.
Received 6 July 1993; and in revised form 2 August 1993.

### Materials and methods

### *Chemicals*

Paclitaxel powder and Cremophor EL were supplied by the Cancer Therapy Evaluation Program (CTEP), National Cancer Institute. The powder was dissolved in dimethyl sulfoxide (DMSO, Sigma Chemical Co., St Louis, MO.) to a stock concentration of 10 mM. Paclitaxel formulated in Cremophor EL was obtained from the Pharmacy Branch of the Clinical Center at the NIH at a stock concentration of 6 mg $ml^{-1}$ (7.04 mM). Unless specifically noted otherwise, all studies used paclitaxel formulated in Cremophor EL.

### *Cell culture*

All cell lines used in these studies were of human origin. The breast adenocarcinoma MCF-7, lung adenocarcinoma A549, cervical carcinoma HeLa, grade III astrocytoma U373, and colon adenocarcinoma HT-29 cell lines were all obtained from ATCC (Rockville, MD). The ovarian adenocarcinoma OVG-1 cell line was established in the laboratory of the Radiation Oncology Branch from patient material taken at the Clinical Center of the NIH. The pancreatic adenocarcinomas PC-Sh and PC-Zd cell lines were established and kindly provided to us by Dr William Sindelar of the Surgery Branch from surgical specimens taken at the Clinical Center of the NIH. MCF-7, A549, HeLa, HT-29, and OVG-1 were all maintained in RPMI 1640 medium supplemented with 10% foetal bovine serum (FBS) and antibiotics. U373, PC-Sh, and PC-Zd were grown in Dulbecco's modified minimal essential medium (DMEM) supplemented with 20% FBS and antibiotics. For cell survival experiments, a number of 100 mm petri dishes were plated with $5 \times 10^5$ cells. Exponentially growing cells were exposed to various concentrations of paclitaxel or its appropriate diluent 24 h later. For experiments that studied cells in plateau phase of growth, cells were permitted to grow for a minimum of 72 h before they were exposed to paclitaxel. Cell counts of cultures grown in parallel confirmed no net growth of cells in cultures used in plateau phase experiments. Additionally, DNA flow cytometry studies demonstrated that 75–80% of cells in plateau phase cultures were in G1, compared to only 45-47% of cells in exponentially growing cultures. After exposure to paclitaxel for various times, the cells were rinsed, trypsinised, and washed with medium. The cells were re-suspended as single cell suspensions, counted with a particle counter (Elzone® 180XY; Particle Data Inc., Elmhirst, IL), plated, and incubated for macroscopic colony formation. Following a one to two week incubation, colonies were fixed with methanol/acetic acid (3:1), stained with crystal violet, and colonies with > 50 cells counted. All survival points were done in triplicate and experiments were conducted a minimum of two times. Error bars shown in the Figures represent s.e.m. and are shown when larger than the symbol. Plating efficiencies for cells were in the following ranges: MCF-7, 45–60%; A549, 40–50%; OVG-1, 40–50%; PC-Sh, 40–50%; PC-Zd, 40–50%; HeLa, 75–85%; U373, 15–25%; and HT-29, 35–45%. Plating efficiencies of all cell lines used in these studies were unaffected by cell density.

### Results

### *Cell survival*

All exponentially growing human tumour cell lines exposed for 24 h to paclitaxel formulated in Cremophor EL exhibited characteristic dose-response curves (Figures 1 and 2). These curves were distinguished by an initial steep decline in cell survival such that the $IC_{50}$ for most of the cell lines ranged between paclitaxel concentrations of 2.5 nM and 7.5 nM (Table I). Above paclitaxel concentrations of 50 nM, however, survival remained constant in all cell lines. No additional cell killing was observed in any of the cell lines exposed to paclitaxel for 24 h when paclitaxel concentrations ranged from 50 nM to 10,000 nM (200 fold range in concentration). Several cell lines showed an *enhanced* cell survival when the concentration of paclitaxel was 10,000 nM. This improvement in cell survival at very high concentrations of paclitaxel was most marked in the A549 cell line.

Because the possibility existed that the enhancement of survival seen at 10,000 nM paclitaxel was due to the large amount of Cremophor EL (0.135% v/v) added to the cells, A549 and MCF-7 cells were exposed to relatively low concentrations of paclitaxel (≤20 nM) for 24 h (Figure 2). As noted previously, cells showed a sharp decline in survival with increasing concentrations of paclitaxel up to 20 nM. However, cells incubated in the same low paclitaxel levels in the presence of 0.135% v/v Cremophor EL – the same concentration of Cremophor El present in a solution of 10,000 nM paclitaxel – showed a marked reduction in cytotoxicity. A similar set of experiments in which cell lines were exposed to paclitaxel in high levels of DMSO (0.135% v/v) showed no effect of DMSO on paclitaxel cytotoxicity (data not shown). Neither diluent alone had any effect on cell survival at concentrations up to 0.135% v/v (note that plating efficiency of cells in Figure 2 is unaffected by Cremophor EL; data for DMSO are not shown). Further evidence of the ability of high levels of Cremophor EL to abrogate paclitaxel cytotoxicity in A549 cells is shown in Figure 3. A549 cells were treated with increasing concentrations of paclitaxel that had been dissolved in DMSO. DMSO did not alter either the initial decline in cell survival at low concentrations of paclitaxel or the uniform cytotoxicity that occurs between 25 nM and 1000 nM. However, the paradoxical increase in survival seen at very high concentrations of paclitaxel was affected by the choice of diluent. No increase in survival in A549 cells was seen at 10,000 nM paclitaxel, when the drug was dissolved in DMSO.

As dose-response curves for 24 h paclitaxel exposure demonstrated little effect of increased concentration on cytotoxicity, studies were carried out to examine the effect of prolonged paclitaxel exposure on cell survival. Cells were incubated in 5 or 50 nM paclitaxel for periods ranging from 6 to 72 h. Figure 4 shows that cells suffered little or no cytotoxicity when exposed to paclitaxel for only 6 h, and greatly reduced cytotoxicity after 12 h compared to 24 h of paclitaxel treatment. Further exposure to paclitaxel for 48 or 72 h resulted in increased cytotoxicity in cells exposed to 50 nM paclitaxel. The enhancement of cytotoxicity with increasing time of exposure to paclitaxel varied among cell lines; MCF-7 cells had only a 5-fold increase in cell kill while

**Figure 1** Survival of four human tumour cell lines after exposure to paclitaxel for 24 h. Points represent the means of at least three replicates. Error bars are s.e.m.

**Figure 2** Survival of A549 (**a**) and MCF-7 (**b**) cells exposed to paclitaxel for 24 h. As described in Materials and methods, cells were exposed to either paclitaxel diluted in RPMI or paclitaxel diluted in medium to which Cremophor EL had been added to a final concentration of 0.135% (v/v) – the same concentration of Cremophor EL which is present in a 10 μM solution of paclitaxel. Points represent the mean of at least three replicates. Error bars are s.e.m. Plating efficiency for control A549 cells was 43% and for control MCF-7 cells was 60%. Plating efficiency for A549 and MCF-7 cells exposed to 0.135% v/v Cremophor EL was 43% and 57%, respectively.

**Table I** Concentration of paclitaxel required to kill 50% of cells in exponentially growing cultures after a 24 h exposure to the drug

| *Cell lines* | *Tumour type* | *IC50 (nM)* |
|---|---|---|
| HeLa | Cervical carcinoma | 2.6 |
| A549 | Lung adenocarcinoma | 4.1 |
| U373 | Grade III astrocytoma | 4.2 |
| MCF-7 | Breast adenocarcinoma | 2.5 |
| HT-29 | Colon adenocarcinoma | 2.8 |
| OVG-1 | Ovarian carcinoma | 4.0 |
| PC-Sh | Pancreatic adenocarcinoma | 7.5 |
| PC-Zr | Pancreatic adenocarcinoma | 4.0 |

Each value represents the mean of at least two experiments.

**Figure 3** Survival of A549 cells after exposure for 24 h to paclitaxel. Paclitaxel was formulated initially at a concentration of 6 mg ml⁻ in Cremophor EL or DMSO and then diluted in RPMI medium. Points represent the means of at least three replicates. Error bars depict s.e.m.

**Figure 4** Survival of A549 (**a**) MCF-7 (**b**), and PC-Sh (**c**) cells after exposure to 5 nM or 50 nM paclitaxel for up to 72 h. Points represent the means of at least three replicates. Error bars depict s.e.m.

A549 cells had a 2 log increase in cytotoxicity at 72 h compared to 24 h of paclitaxel exposure. In contrast to the enhanced cytotoxicity seen with prolonged incubation of cells in 50 nM paclitaxel, continuous incubation in 5 nM paclitaxel resulted in little additional cell kill after 24 h of exposure.

The issue of the effect of increasing paclitaxel concentration combined with increasing duration of exposure to the drug on cytotoxicity is addressed in Figure 5. Little or no increased cell killing was observed in cell lines exposed to paclitaxel concentrations of more than 50 nM even after 48 or 72 h of drug exposure. The marked increase in cell survival noted after a 24 h exposure to 10,000 nM paclitaxel was maintained at the later time points.

In contrast to exponentially growing cells, cells that were permitted to grow to plateau phase were relatively resistant to paclitaxel (Figure 6). The initial decline in survival seen with increasing paclitaxel concentrations in exponentially growing cultures was altered in plateau phase cells leading to an increase in the $IC_{50}$ for all cell lines. Additionally, the stable nadir in cell survival seen with increasing concentrations of paclitaxel was also markedly increased in plateau phase cells compared with exponentially growing cells.

### Discussion

We have shown, in a variety of different human tumour cell lines, that paclitaxel has a unique dose-response cytotoxic effect. All lines exposed to paclitaxel for 24 h that we have studied thus far exhibit a sharp decline in cell survival at low concentrations of the drug. However, each line also demonstrated a plateau in survival at concentrations of paclitaxel above 50 nM. At a very high concentration of paclitaxel, 10,000 nM, several cell lines showed an increase in cell survival. The increase in cell survival at 10,000 nM paclitaxel may have been due largely to the high concentration of Cremophor EL (0.135% v/v) present. The shape of the dose-response curves observed at paclitaxel concentrations of less than 1000 nM – that is, an initially steep decline in survival followed by a plateau in cytotoxicity at higher doses – has been reported with other drugs, including the vinca alkaloids (Hill & Whelan, 1981). However, we are not aware of any reports showing dramatic increases in survival of cells exposed to increasing amounts of cytotoxic drugs as we have observed in cells exposed to 10,000 nM paclitaxel.

The concentrations of paclitaxel that we have used in these studies are similar to plasma concentrations of the drug that have been achieved in clinical trials (Rowinsky et al., 1990). Brief (one to six hour) infusions of paclitaxel have resulted in peak plasma concentrations of 1000 to 10,000 nM. When paclitaxel has been given over 24 h, peak plasma levels have ranged from 600 to 3500 nM. More recently, paclitaxel steady state plasma levels obtained during a 96 h infusion of the drug have been described (Wilson et al., 1993) and range

**Figure 5** Survival of A549 (**a**) and MCF-7 (**b**) cells after exposure to various concentrations of paclitaxel for 24, 48, or 72 h. Points represent the means of at least three replicates. Error bars depict s.e.m.

**Figure 6** Survival of A549 (**a**) and MCF-7 (**b**) cells after exposure to paclitaxel for 24 h. As described in Materials and methods, either exponentially growing cell cultures in plateau phase of growth were exposed to paclitaxel and survival was assessed by clonogenic assay. Points represent the means of at least three replicates. Error bars are s.e.m.

from 53 to 77 nM. Given that the terminal half-life of paclitaxel in plasma ranges from five to eight hours (Rowinsky *et al.*, 1990), the concentrations and durations of exposure of the drug that we have used *in vitro* approximate what has been achieved clinically in plasma.

Though cells exposed to various concentrations of paclitaxel for a fixed period of time (24 h) showed no increase in cytotoxicity in response to drug concentrations above 50 nM, increasing the time of exposure to paclitaxel did result in a marked increase in cytotoxicity. Little or no cytotoxicity was seen in cells that were treated with paclitaxel for less than 12 h. Cytotoxicity then increased in all cell lines as time of exposure to paclitaxel increased, so that cell killing after 72 h was as much as 200 times greater than that seen after 24 h of paclitaxel treatment. However, even at exposure times of 72 h, little or no additional cytotoxicity was achieved when paclitaxel concentrations were raised above 50 nM. These results show that cytotoxicity due to paclitaxel is very dependent on the duration of exposure to the drug and less dependent on the concentration of paclitaxel to which cells are exposed.

We believe that these findings lead to two conclusions which have profound implications for the clinical use of paclitaxel. First, achieving peak tumour drug levels above 50 nM paclitaxel is unlikely to be rewarded with increased tumour response. Second, extended exposure to paclitaxel is likely to result in a greater tumour response than would be expected from the administration of bolus doses of the drug. These conclusions imply that the optimal delivery of paclitaxel to most patients would be via a prolonged continuous infusion with the goal of achieving steady state drug levels of about 50 nM. It should be stressed that an exact *in vivo* steady state plasma drug level target can not be determined directly from our *in vitro* data – plasma levels may not reflect tumour drug levels. For example, recent pharmacokinetic data (Markman *et al.*, 1992) have shown that intraperitoneal paclitaxel levels are maintained far longer than in plasma. However, in no exponentially growing human tumour cell line that we have studied have we seen enhanced cytotoxicity with paclitaxel concentrations over 50 nM. (N.B., this statement applies only to tumour lines that are not resistant to paclitaxel; we, and others (Horwitz *et al.*, 1986), have found that cells that express the multiple drug resistance phenotype are markedly resistant to high (1000 nM) concentrations of paclitaxel.)

The current data are also consistent with results that have been reported from clinical trials. We have found that paclitaxel is an active agent against a variety of different human tumour cells. However, in the absence of prolonged exposure of cells to paclitaxel, we have found surviving fractions of 0.01 to 0.1 in cells exposed to clinically relevant concentrations of paclitaxel for 24 h. To date, the overwhelming majority of clinical responses reported after the use of single agent paclitaxel have been partial responses and have been of brief – less than one year – duration (McGuire *et al.*, 1989; Holmes *et al.*, 1991). Although overall response rates in clinical trials of paclitaxel in breast and ovarian cancer have been impressive, the large proportion of partial responses and the brief nature of most responses suggest that a considerable amount of viable tumour remains after single agent paclitaxel therapy.

Non-proliferating cells were markedly more resistant to paclitaxel than were cells growing exponentially. Similar findings have recently been reported in N417 small cell lung cancer cells (Riou *et al.*, 1992). It has been recognised for some time that cell cycle specific chemotherapeutic agents are less toxic to non-proliferating cells (Drewinko *et al.*, 1981) and paclitaxel fits this pattern very well. It is possible that plateau phase cells that are exposed to paclitaxel for longer durations would suffer more cytotoxicity than we found after a 24 h exposure to the drug. However, because of the difficulty of maintaining cells in plateau phase in culture for prolonged periods of time, we were unable to test the hypothesis that prolonged exposure to paclitaxel increases the killing of plateau phase cells in our *in vitro* systems.

Cremophor EL, the diluent in which paclitaxel is prepared for clinical use, has been shown to be a protein kinase C inhibitor in cell extracts (Chaung *et al.*, 1991) and may be able to reverse the multiple drug resistance phenotype (Woodcock *et al.*, 1990). Our studies confirm that this diluent has biologic effects, but suggest that high levels of Cremophor can antagonise the cytotoxicity of paclitaxel. Concentrations of Cremophor EL, equivalent to that which would be present in a 10,000 nM solution of paclitaxel, inhibited the cytotoxicity normally seen after cells are exposed to paclitaxel for 24 h (Figure 2). High levels of DMSO did not alter paclitaxel cytotoxicity. A possible explanation for the antagonistic effect of high levels of Cremophor EL on paclitaxel cytotoxicity can be found in DNA flow cytometry studies of A549 cells (Liebmann *et al.*, 1993*b*). We and others (Schiff & Horwitz, 1980) have found that exposure of exponentially dividing cells to paclitaxel rapidly results in a block in the G2/M phases of the cell cycle. However, a significant fraction of A549 cells exposed to paclitaxel in a high concentration of Cremophor EL remain in G1 instead of progressing to a block in G2/M (Liebmann *et al.*, 1993*b*). If Cremophor EL is able to produce a G1 block, then cells would be unable to enter G2 and M where the cytotoxic effect of paclitaxel is apparently manifest. We have seen a similar antagonistic effect of paclitaxel cytotoxicity with glutathione depletion by L-buthionine sulfoximine in A549 cells (Liebmann *et al.*, 1993*a*).

It is unclear whether the *in vitro* conditions involving high concentrations of Cremophor EL would be replicated *in vivo*. There is considerable information about the pharmacokinetics of paclitaxel from Phase I trials (Rowinsky *et al.*, 1990). Little is known about the pharmacokinetics of Cremophor EL, but it seems unlikely that this diluent would persist in plasma for a long period of time. Recently, however, there has been a report of the intraperitoneal administration of paclitaxel (Markman *et al.*, 1992). In that study extraordinarily high concentrations of paclitaxel (> 100,000 nM) were obtained in the peritoneal space. If Cremophor EL is not rapidly cleared from the peritoneum it may exert considerable effects, possibly including the antagonism of paclitaxel cytotoxicity. Though this would be of concern for the intra- peritoneal administration of paclitaxel, it would not be unsurmountable. The half-life of paclitaxel is markedly prolonged in the peritoneum (Markman *et al.*, 1992). Lower doses of paclitaxel which resulted in lower peak levels of paclitaxel ($\leqslant$1000 nM) would still result in an extended exposure of tumour to paclitaxel. Because of the time dependency of paclitaxel cytotoxicity, this should provide excellent tumour kill while avoiding potential problems from high concentrations of Cremophor EL.

In summary, we have shown, with *in vitro* clonogenic assays, that the dose-response curve of paclitaxel cytotoxicity is initially steep at low concentrations of the drug, but then is flat over a wide range of paclitaxel concentrations. Further, at a very high concentration of paclitaxel (10,000 nM), cell survival significantly increases compared with survival at lower paclitaxel concentrations. By contrast, increasing the time of paclitaxel exposure results in increasing paclitaxel cytotoxicity. Cells in plateau phase of growth are much more resistant to paclitaxel than are exponentially growing cells. High levels of Cremophor EL, the diluent in which paclitaxel is prepared for clinical use, can antagonise paclitaxel cytotoxicity and may be responsible for the increase in cell survival noted in cells exposed to 10,000 nM paclitaxel. These results suggest that paclitaxel will be most effective clinically if it is administered to patients with the goal of maintaining modest plasma levels (about 50 nM) for several days.

### References

CARMICHAEL, J., DEGRAFF, W.G., GAZDAR, A.F., MINNA, J.D. & MITCHELL, J.B. (1987). Evaluation of a tetrazolium based semiautomated colorimetric assay: I. Assessment of chemosensitivity testing. *Cancer Res.*, **47**, 936–942.

CHANG, A., KIM, K., GLICK, J., ANDERSON, T., KARP, D. & JOHNSON, D. (1992). Phase II study of taxol in patients with stage IV non-small cell lung cancer (NSCLC): The Eastern Cooperative Oncology Group (ECOG) results. *Proc. Am. Soc. Clin. Oncol.*, **11**, 293.

CHAUNG, L.F., ISRAEL, M. & CHUANG, R.Y. (1991). Cremophor EL inhibits 12-*O*-tetradecanoylphorbol-13 acetate (TPA)- induced protein phosphorylation in human myeloblastic leukemia ML-1 cells. *Anticancer Res.*, **11**, 1517–1521.

COOK, J.A. & MITCHELL, J.B. (1989). Viability measurements in mammalian cell systems. *Analytical Biochem.*, **179**, 1–7.

COOK, J.A., LIEBMANN, J., SULLIVAN, F., HAHN, S., TEAGUE, D., DEGRAFF, W. & MITCHELL, J.B. (1993). Paclitaxel mediated cytotoxicity in Chinese hamster V79 cells. *Cancer Chemother. Pharmacol.*, (Accepted for publication)

DREWINKO, B., PATCHEN, M., YANG, L.Y. & BARLOGIE, B. (1981). Differential killing efficacy of twenty antitumor drugs on proliferating and nonproliferating human tumor cells. *Cancer Res.*, **41**, 2328–2333.

HILL, B.T. & WHELAN, R.D.H. (1981). Comparative cell killing and kinetic effects of vincristine or vindesine in mammalian cell lines. *J. Natl Cancer Inst.*, **67**, 437–443.

HOLMES, F.A., WALTERS, R.S., THERIAULT, R.L., FORMAN, A.D., NEWTON, L.K., RABER, M.N., BUZDAR, A.U., FRYE, D.K. & HORTOBAGYI, G.N. (1991). Phase II trial of taxol, an active drug in the treatment of metastatic breast cancer. *J. Natl Cancer Inst.*, **83**, 1797–1805.

HORWITZ, S.B., LOTHSTEIN, L., MANFREDI, J.J., MELLADO, W., PARNESS, J., ROY, S.N., SCHIFF, P.B., SORBARA, L. & ZEHEB, R. (1986). Taxol: Mechanisms of action and resistance. *Ann. N.Y. Acad. Sci.*, **466**, 733–744.

LIEBMANN, J.E., HAHN, S.M., COOK, J.A., LIPSCHULTZ, C.A., MITCHELL, J.B. & KAUFMAN, D.C. (1993*a*). Glutathione depletion by L-buthionine sulfoximine antagonizes taxol cytotoxicity. *Cancer Res.*, **53**, 2066–2070.

LIEBMANN, J., COOK, J.A., LIPSCHULZ, C., TEAGUE, D., FISHER, J. & MITCHELL, J.B. (1993*b*). Antagonism of paclitaxel by Cremophor EL in human tumor cells. *Cancer Chemother. Pharmacol.*, (accepted for publication).

MANFREDI, J.J. & HORWITZ, S.B. (1984). Taxol: An antimitotic agent with a unique mechanism of action. *Pharmacol. Ther.*, **25**, 83–125.

MARKMAN, M., ROWINSKY, E., HAKES, T., REICHMAN, B., JONES, W., LEWIS, Jr. J.L., RUBIN, S., CURTIN, J., BARAKAT, R., PHILLIPS, M., HUROWITZ, L., ALMADRONES, L. & HOSKINS, W. (1992). Phase I trial of intraperitoneal taxol: A Gynecologic Oncology Group study. *J. Clin. Oncol.*, **10**, 1485–1491.

MCGUIRE, W.P., ROWINSKY, E.K., ROSENSHEIN, N.B., GRUMBINE, F.C., ETTINGER, D.S., ARMSTRONG, D.K. & DONEHOWER, R.C. (1989). Taxol: A unique antineoplastic agent with significant activity in advanced ovarian epithelial neoplasms. *Ann. Intern Med.*, **111**, 273–279.

RIONDEL, J., JACROT, M., PICOT, F., BERIEL, H., MOURIQUAND, C. & POTIER, P. (1986). Therapeutic response to taxol of six human tumors xenografted into nude mice. *Cancer Chemother. Pharmacol.*, **17**, 137–142.

RIOU, J.-F., NAUDIN, A. & LAVELLE, F. (1992). Effects of taxotere on murine and human tumor cell lines. *Biochem, Biophys. Res. Commun.*, **187**, 164–170.

ROWINSKY, E.K., DONEHOWER, R.C., JONES, R.J. & TUCKER, R.W. (1988). Microtubule changes and cytotoxicity in leukemic cell lines treated with taxol. *Cancer Res.*, **48**, 4093–4100.

ROWINSKY, E.K., BURKE, P.J., KARP, J.E., TUCKER, R.W., ETTINGER, D.S. & DONEHOWER, R.C. (1989). Phase I and pharmacodynamic study of taxol in refractory acute leukemias. *Cancer Res.*, **49**, 4640–4647.

ROWINSKY, E.K., CAENAVE, L.A. & DONEHOWER, R.C. (1990). Taxol: A novel investigational antimicrotubule agent. *J. Natl Cancer Inst.*, **82**, 1247–1259.

SCHIFF, P.B., FANT, J. & HORWITZ, S.B. (1979). Promotion of microtubule assembly *in vitro* by taxol. *Nature*, **277**, 665–667.

SCHIFF, P.B. & HORWITZ, S.B. (1980). Taxol stabilizes microtubules in mouse fibroblast cells. *Proc. Natl Acad. Sci. USA*, **77**, 1561–1565.

WILSON, W.H., BERG, S., KANG, Y.-K., BATES, S., FOJO, A., STEINBERG, S., BALIS, F., GOLDSPIEL, B., O'SHAUGHNESSY, J., CHABNER, B. & WITTES, R.E. (1993). Phase I/II study of taxol 96-hour infusion in refractory lymphoma and breast cancer: pharmacodynamics and analysis of multi-drug resistance (*mdr*1). (abstract #335). *Proc. Amer. Soc. Clin. Oncol.*, **12**, 134.

WOODCOCK, D.M., JEFFERSON, S., LINSENMEYER, M.E., CROWTHER, P.J., CHOJNOWSKI, G.M., WILLIAMS, B. & BERTONCELLO, I. (1990). Reversal of the multidrug resistance phenotype with Cremophor EL, a common vehicle for water-insoluble vitamins and drugs. *Cancer Res.*, **50**, 4199–4203.